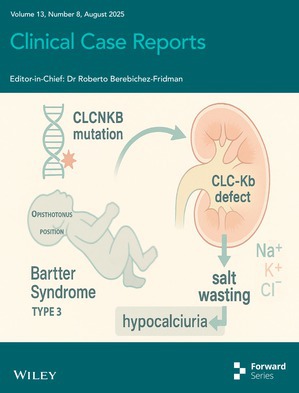# Cover Image

**DOI:** 10.1002/ccr3.70831

**Published:** 2025-09-02

**Authors:** Karim Hassan, Imad Afara, Ali Harajli, Jawad Allam, Kaity Saliba, Frederic Harb

## Abstract

The cover image is based on the article *An Atypical Presentation of Bartter Syndrome Type 3 With Hypocalciuria and Opisthotonus Posture in a Preterm Infant* by Karim Hassan et al., https://doi.org/10.1002/ccr3.70725.